# Predicting bankruptcy of firms using earnings call data and transfer learning

**DOI:** 10.7717/peerj-cs.1134

**Published:** 2023-01-04

**Authors:** Hafeez Ur Rehman Siddiqui, Beatriz Sainz de Abajo, Isabel de la Torre Díez, Furqan Rustam, Amjad Raza, Sajjad Atta, Imran Ashraf

**Affiliations:** 1 Faculty of Computer Science and Information Technology, Khawaja Fareed University of Engineering and Information Technology, Rahim Yar Khan, Pakistan; 2 Department of Signal Theory, Communications and Telematics Engineering, Unviersity of Valladolid, Spain; 3 School of Computer Science, University College Dublin, Dublin, Ireland; 4 Department of Information and Communication Engineering, Yeungnam University, Gyeongsan si, Republic of Korea

**Keywords:** Bankruptcy prediction, Transfer learning, Feature extraction, Deep learning

## Abstract

Business collapse is a common event in economies, small and big alike. A firm’s health is crucial to its stakeholders like creditors, investors, partners, *etc.* and prediction of the upcoming financial crisis is significantly important to devise appropriate strategies to avoid business collapses. Bankruptcy prediction has been regarded as a critical topic in the world of accounting and finance. Methodologies and strategies have been investigated in the research domain for predicting company bankruptcy more promptly and accurately. Conventionally, predicting the financial risk and bankruptcy has been solely achieved using the historic financial data. CEOs also communicate verbally *via* press releases and voice characteristics, such as emotion and tone may reflect a company’s success, according to anecdotal evidence. Companies’ publicly available earning calls data is one of the main sources of information to understand how businesses are doing and what are expectations for the next quarters. An earnings call is a conference call between the management of a company and the media. During the call, management offers an overview of recent performance and provides a guide for the next quarter’s expectations. The earning calls summary provided by the management can extract CEO’s emotions using sentiment analysis. This article investigates the prediction of firms’ health in terms of bankruptcy and non-bankruptcy based on emotions extracted from earning calls and proposes a deep learning model in this regard. Features extracted from long short-term memory (LSTM) network are used to train machine learning models. Results show that the models provide results with a high score of 0.93, each for accuracy and F1 when trained on LSTM extracted feature from synthetic minority oversampling technique (SMOTE) balanced data. LSTM features provide better performance than traditional bag of words and TF-IDF features.

## Introduction

Business failure is a common event in all economies, small and big alike. Several stakeholders are interested in the well-being of a firm like investment institutions, shareholders, partners, and even customers and suppliers. Therefore prediction of the upcoming financial crisis is significantly important to devise appropriate strategies to avoid business collapses (Horváthová & Mokrišová, 2018). Bankruptcy forecasting has long been an important concern and critical task in accounting and finance (Qu et al., 2019). The bankruptcy prediction models function as warnings and reveal vulnerabilities of financial institutions and provide timely input to managers, investors, and other stakeholders (Kirkos, Spathis & Manolopoulos, 2007). Several efforts have laid down the foundations to create systems and strategies for predicting company bankruptcy more promptly and accurately (Barboza, Kimura & Altman, 2017). Because of the significant individual, economic, and societal consequences associated with company failures or bankruptcies, attempts have been made to give better insight into and forecast bankruptcy events (McKee & Lensberg, 2002). Up until the mid-twentieth century, there were no advanced statistical tools or computers available to predict bankruptcy. The sole instrument for predicting a company’s bankruptcy was the financial ratios of failing and non-failing firms. (Yoon & Kwon, 2010). The tools used back then were based on statistical approaches such as discriminant analysis (DA) (Horváthová & Mokrišová, 2018) and logistic regression (LR) for bankruptcy prediction (McKee & Lensberg, 2002). Non-parametric methods like decision trees (DT) appeared in the 1980s (Yoon & Kwon, 2010). Machine learning (ML) classifiers such as DT, neural networks (NN), and support vector machines (SVM) have been frequently employed to forecast company failure since the 1990s (Christy & Arunkumar, 2019).

Predominantly, predicting financial risk and bankruptcy has been following the historic financial data where different financial ratio-based indicators and variables are considered for bankruptcy prediction (Kregar, 2011). Several studies focus on using financial indicators and business environment factors to increase the prediction accuracy (Zelenkov & Volodarskiy, 2021). However, such information is not reliable due to the lack of internal control. In addition, financial ratios and other financial markers show a firm’s performance for the previous year and do not present its current status of operations (Balcaen & Ooghe, 2006). Predominantly the emphasis is placed on the optimization of the predicting models by joining multiple models or by refining the data following preprocessing steps like data cleaning, normalization, *etc.*, (Muslim & Dasril, 2021; Jaki & Ćwiek, 2021).

Recently, several papers on finance and finance management explored the possibility of using textual data for financial risk prediction. For this purpose, the sentiment related to financial disclosures can be quantified and used for risk prediction. Business meetings involving investors and top management for discussing a firm’s financial position are nonverbal ways that CEOs communicate today. CEOs also communicate verbally *via* press releases. Voice characteristics, such as emotion and tone, may reflect a company’s current status according to anecdotal evidence (Qin & Yang, 2019). The extent to which vocal features may be used to forecast danger levels, however, is not yet fully established. The use of top management sentiments and textual cues for risk prediction increased the interest in the natural language processing (NLP) field as well. For example, several research articles have used text-based techniques to predict volatility (Wang & Hua, 2014). Such approaches are specifically useful in scenarios lacking appropriate financial disclosures. This study leverages the text data for predicting the bankruptcy of a firm and makes the following contributions

• This study gathers a large dataset comprising the data of 1,016 companies from the ‘Seeking Alpha’ website. The data is collected for different individual companies, structured, preprocessed, and manually labeled.
• A novel transfer learning-based model is proposed where the long short-term memory (LSTM) is used for feature extraction to train the machine learning models for training and testing. LSTM is custom designed for this purpose.
• Several machine learning models are also evaluated including extra tree classifier (ETC), AdaBoost (ADA), random forest (RF), logistic regression (LR), and support vector classifier (SVC). In addition, term frequency-inverse document frequency (TF-IDF) and bag of words (BoW) are adopted for machine learning models. The impact of the synthetic minority oversampling technique (SMOTE) is also investigated.

This article is divided into five sections. Section ‘Related Work’ contains the literature related to the problem at hand. It is followed by a description of the proposed approach, feature extraction, and ensemble method. Afterward, the results are discussed. In the end, the study is concluded in the last section.

## Related Work

Given the fundamental changes associated with globalization, more accurate predicting of company financial troubles would be important for decision-makers such as investors, creditors, government officials, and even the general public. Business bankruptcies can be triggered by a variety of causes, including poor investment decisions, a weak investment climate, insufficient cash flow, and so on (Yoon & Kwon, 2010). As a result, the numerous present approaches for forecasting business failure must be constantly improved.

Several researchers have used artificial intelligence (AI) approaches to forecast bankruptcy during the last decade. For example, a bankruptcy prediction based on artificial neural network (ANN) and LR models is presented in Kasgari, Salehnezhad & Ebadi (2013). The data of 120 manufacturing companies including 54 bankrupts and 66 non-bankrupts listed on the Tehran Stock Exchange is used for experiments. Financial ratios, such as the current, quick ratio and return-on-asset ratio, debt ratio, and the ratio of operating capital to total assets are used to predict bankruptcy. Results indicate that the probability values of bankruptcy prediction one year before bankruptcy are 87.9% and 90.7% for non-bankrupt and bankrupt enterprises, respectively using the LR model. While bankrupt and non-bankrupt companies are predicted to be 90.9% and 90.7% respectively, using the ANN model. Analysis shows that the designed ANN model has distinguished between the bankrupt and non-bankrupt firms with higher accuracy.

Business meetings involving investors and earnings conference calls are two nonverbal ways that CEOs communicate today. Voice characteristics, such as emotion and tone, may reflect a company’s success, according to anecdotal evidence, and can be used to predict the bankruptcy of a firm. For example, Wang et al. (2013) analyzes the influence of sentiment words related to financial situations. Such terms are used in financial reports and contain sentiments related to the firm’s financial disclosure. The study applied regression techniques to the finance sentiment lexicon and investigate the possibility of predicting financial risk using sentiment words. Experimental results using BoW features and ranking models show that the results are promising with comparable performance. It indicates that the financial sentiment words can be leveraged to perform risk analysis and bankruptcy analysis as a high correlation is found between sentiment words and the risk of bankruptcy.

A deep learning technique for recognizing significant financial events from the text and extracting natural language is studied in Rönnqvist & Sarlin (2017). The modeling is based on the use of entities and chronology to link the text and event data. The event data is collected for 101 large European banks during three phases of the financial crisis from 2007 to 2009. Several financial events are considered for this purpose like government and state aid, company failures, and destabilized mergers, while the text data includes news items from the Reuters online archive for 2007 to 2014 (Q3). Deep neural networks are used to predict events from text and are trained in two steps. The first step is pre-training using sentence vectors while supervised models are used to learn events in the second step. To train and deploy the model, the semantic pre-training stage produces sentence vectors for each of the 716 k sentences. A typical distress prediction tool, the relative usefulness metric, is used to evaluate the model’s performance. It provides a more accurate prediction of future performance when the algorithm is applied to unknown data or banks. When banks aggregate, performance increases by 12.3%, whereas vector-level assessments have a usefulness of 8.3%.

Qin & Yang (2019) obtained a dataset comprised of earning call transcripts and corresponding earning call audios obtained from the website ‘Seeking Alpha’ and ‘EarningsCast’, respectively. An iterative forced alignment (IFA) technique is used that aligns each sentence of the transcript with the audio clip containing the sentence’s spoken text. To extract textual features, the arithmetic mean of each sentence represented as a 300-dimension vector is calculated by embedding GloVe-300 that is pre-trained on Wikipedia and Gigaword. Praat is used to extract 27 audio features from audio recordings, including pitch, intensity, *etc.* The multimodal deep regression model (MDRM) is used with two components where the first component is a contextual BiLSTM while the second component is the training model. The first component extracts unimodal features from either text or audio. The collected multimodal (text and audio) features are then merged in the second component and fed to a BiLSTM with a fully connected layer, which extracts interdependencies between text and audio modalities. Results suggest that MDRM outperforms all baseline models when using both text and audio data, with prediction errors of 1.371, 0.420, 0.300, and 0.217 for the three, seven, fifteen, and thirty days following the conference call, respectively.

Predominantly, predicting and analyzing financial risk utilize quantitative data from financial documents and surveys. Research efforts that follow alternative solutions are very limited. For example, Nopp & Hanbury (2015) investigates the feasibility of sentiment analysis to predict the financial risk using the opinions and sentiment words from the text data. For this purpose, letters from CEOs and important data from banks’ periodic reports are utilized. A lexicon-based approach and a supervised risk prediction model are used. The former approach involves analyzing the sentiments from published reports like negative and positive words, and words representing uncertainty while the latter predicts factors employing machine learning classification. For example, a capital ratio (T1) is used that indicates the strength of a bank to bear losses. Predictions are relatively inaccurate at the level of individual banks. Conversely, using aggregated figures show a strong relationship between the negative or uncertainty showing words in financial reports and bankruptcy or financial crisis.

Theoretical results in Kristóf & Virág (2020) state that no bankruptcy forecasting model can function excluding time, geography, *etc.* Appropriate data preparation and data transformation methods significantly improve model prediction capacity. Predicting market volatility is critical for financial risk within the context of investment decisions. Despite the reports regarding the usefulness of natural language information to enhance the performance of purely financial models, it is still comparably underexplored. Theil, Broscheit & Stuckenschmidt (2019) introduces PRoFET which is a neural specifically designed to predict volatility. It exploits semantic language content and several important financial indicators like past volatility (Kogan et al., 2009), book-to-market, earning surprise (Price et al., 2012), *etc.* As language data, earning calls data from company quarterly meetings is used. Ninety Thousand earnings call transcripts are collected that cover 4.3 K distinct Canadian companies for the period of 2002 to 2017. For handling the bias, a dataset is split into 0.8, 0.1, and 0.1 ratios for train, testing, and validation, respectively. Results confirm the superior performance of the joint model that uses both feature types alone. In addition, the impact of different document sections is also analyzed. Results also show that modeling the verbal context can lead to better results than existing models and baseline models.

Two recent efforts in the context of financial risk prediction are Yang et al. (2020) and Li et al. (2020). The authors propose a hierarchical transformer-based learning model to predict the volatility of financial institutions (Yang et al., 2020). For this purpose, text and audio data of quarterly earning calls are used for volatility prediction. The authors conclude that the use of earning calls data provide useful features for accurate volatility prediction and can be used for related tasks such as asset pricing, fraud detection, *etc.* Li et al. (2020) introduces the earning calls data for S&P 1500 companies. The study discusses the challenges of text and audio alignment and performs volatility prediction using earnings call data.

A comparative analysis of the discussed research works is presented in Table 1. Keeping in view the scope and potential of earning calls data, this study integrates the data with a custom-designed transfer learning framework to achieve higher accuracy for bankruptcy prediction.

**Table 1:** Comparative summary of discussed research works.

**Ref.**	**Year**	**Title**	**Contribution**
Goletsis et al. (2012)	2012	Bankruptcy Prediction through Artificial Intelligence	Applied radial basis function neural networks and the support vector machines are applied to the bankruptcy problem.
Garcia-Almanza, Alexandrova-Kabadjova & Martinez-Jaramillo (2013)	2013	Bankruptcy prediction for banks: An artificial intelligence approach to improve understandability	Used multi-population evolving decision rules MP-EDR to determine the relevance of some features from federal deposit insurance corporation (FDIC) data to predict bank bankruptcy.
Lee & Choi (2013)	2013	A multi-industry bankruptcy prediction model using back-propagation neural network and multivariate discriminant analysis	Investigation of the bankruptcy of Korean companies using back-propagation neural network (BNN) is performed in this research.
Yu et al. (2014)	2014	Bankruptcy prediction using extreme learning machine and financial expertise	Combo method and further ensemble model is investigated based on different LOO-IELM models and the specific financial indicators and leave-one-out-incremental extreme learning machine (LOO-IELM) are explored for predict bankruptcy.
Hájek & Olej (2015)	2015	Word Categorization of Corporate Annual Reports for Bankruptcy Prediction by Machine Learning Methods	Used neural networks, support vector machines, decision trees, and ensembles of decision trees to predict corporate bankruptcy.
Kim & Ahn (2015)	2015	A Hybrid Under-sampling Approach for Better Bankruptcy Prediction	Used data from H Bank’s non-external auditing companies in Korea and compared the performances of the classifiers with the proposed under-sampling and random sampling data.
Slavici, Maris & Pirtea (2016)	2016	Usage of artificial neural networks for optimal bankruptcy forecasting. Case study: Eastern European small manufacturing enterprises	Used ANNs for bankruptcy forecasting.
Walczak (2016)	2016	Artificial neural networks and other AI applications for business management decision support.	This article examines how various ANN and other AI applications may be adapted to facilitate managerial leadership, improve manager performance, and in some cases perform management activities.
Li & Wang (2017)	2017	Machine learning methods of bankruptcy prediction using accounting ratios	Compared the statistical method and machine learning method to predict bankruptcy with utilizing China listed companies and achieved 95% accuracy.
Kim, Mun & Bae (2018)	2018	Data depth based support vector machines for predicting corporate bankruptcy.	Proposed a hybrid method that combines data depths and nonlinear SVM for the prediction of corporate bankruptcy.
Naidu & Govinda (2018)	2018	Bankruptcy prediction using neural networks.	Used ANNs for bankruptcy forecasting.
Nguyen & Pham (2018)	2018	Predicting bankruptcy using machine learning algorithms	Using qualitative bankruptcy dataset, the Random tree bagging (RTree-bagging) ensemble model achieved the highest accuracy with 96.2%.
Islam et al. (2019)	2019	Infusing domain knowledge in AI-based ”black box” models for better explainability with application in bankruptcy prediction	Investigated 33 research papers related to mortgage default/bankruptcy prediction and collected all explanatory variables and applied machine learning methods SVM, ANN, RF, ETC, and GB models for bankruptcy prediction.
Lee, Choi & Oh (2019)	2019	Predicting Corporate Bankruptcy Using Media Information	Used ‘word2vec’ to quantitatively analyze the relationship between the words mentioned in news articles and used logistic regression for bankruptcy prediction.
Kovacova et al. (2019)	2019	Systematic review of variables applied in bankruptcy prediction models of Visegrad group countries	The analysis of more than 100 bankruptcy prediction models developed in V4 countries confirms that enterprises in each country prefer different explanatory variables.
Mai et al. (2019)	2019	Deep learning models for bankruptcy prediction using textual disclosures.	Constructed a comprehensive bankruptcy database of 11,827 U.S. public companies and show that deep learning models yield superior prediction performance in forecasting bankruptcy using textual disclosures.
Chen & Tsai (2020)	2020	Bankruptcy Study Using Artificial Intelligence	Used UCI data bank for prediction of bankruptcy using AI models DT, LR, SVM.
Alam (2021)	2021	Corporate bankruptcy prediction: Analysis of statistical and machine learning models using accounting, market, market microstructure, and derivative instrument information	Used COMPUSTAT, CRSP, Supplemental Short Interest File, S&P Capital IQ, Audit Analytics, and MARKIT databases and used statistical software from Salford Systems, Machine learning model for prediction of Bankruptcy in which ML provided best results.

## Materials and Methods

### Proposed methodology

**Figure 1:**
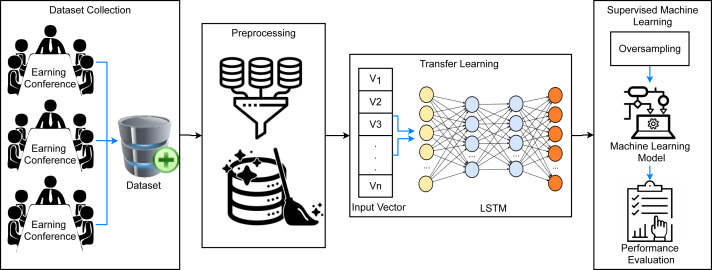
The architecture of the proposed methodology.

This study aims at obtaining a high prediction accuracy for firm’s bankruptcy using the transfer learning-based framework. Figure 1 shows the proposed methodology for the classification of bankrupt and non-bankrupt companies using a supervised machine learning approach. A dataset from different companies earning conferences is collected that contains a large amount of raw data which can create complexity in the training of machine learning models. For this, several preprocessing techniques are used such as conversion to lowercase, stopwords removal, stemming, *etc.* These preprocessing steps remove meaningless data from the dataset and reduce its complexity. Afterward, feature extraction is carried out using recurrent application LSTM which provides significant features to the modes as compared to traditional feature extraction techniques. It is followed by data oversampling using SMOTE which generates synthetic data samples for the minority class (bankrupt) and makes the dataset balanced. Then data is split into training and testing sets in ratios of 0.8 to 0.2 and the performance is evaluated in terms of accuracy, precision, recall, and F1 score.

#### Data collection

Arranging the dataset for analysis of various companies is an important part of the research. For this research, the dataset is collected from the ‘Seeking Alpha’ website. We collected the earning calls data for 1,016 companies from 2008 to 2020. Table 2 shows the sample texts for collected data.

**Table 2:** Sample records from the dataset.

**Title**	**Text**	**Label**
Oasis Petroleum, Inc. (NASDAQ:OAS) Delaware Basin Acquisition Conference Call December 11, 2017 9:15 AM ET	So, I think we’ll continue to look for opportunities to build on this and like I said, I would kind of look at the Williston as a road map, …,	0
Destination Maternity Corporation (NASDAQ:DEST) Q3 2017 Earnings Conference Call December 7, 2017 9:00 AM ET	At this time, all participants are in a listen-only mode. Later we will conduct a question-and-answer session, and instructions will follow at that time, …,	1
Layne Christensen Company (NASDAQ:LAYN) Q3 2018 Results Earnings Conference Call December 6, 2017 9:00 AM ET	Jack Lascar It is now my pleasure to introduce your host, Jack Lascar, …,	0

**Figure 2:**
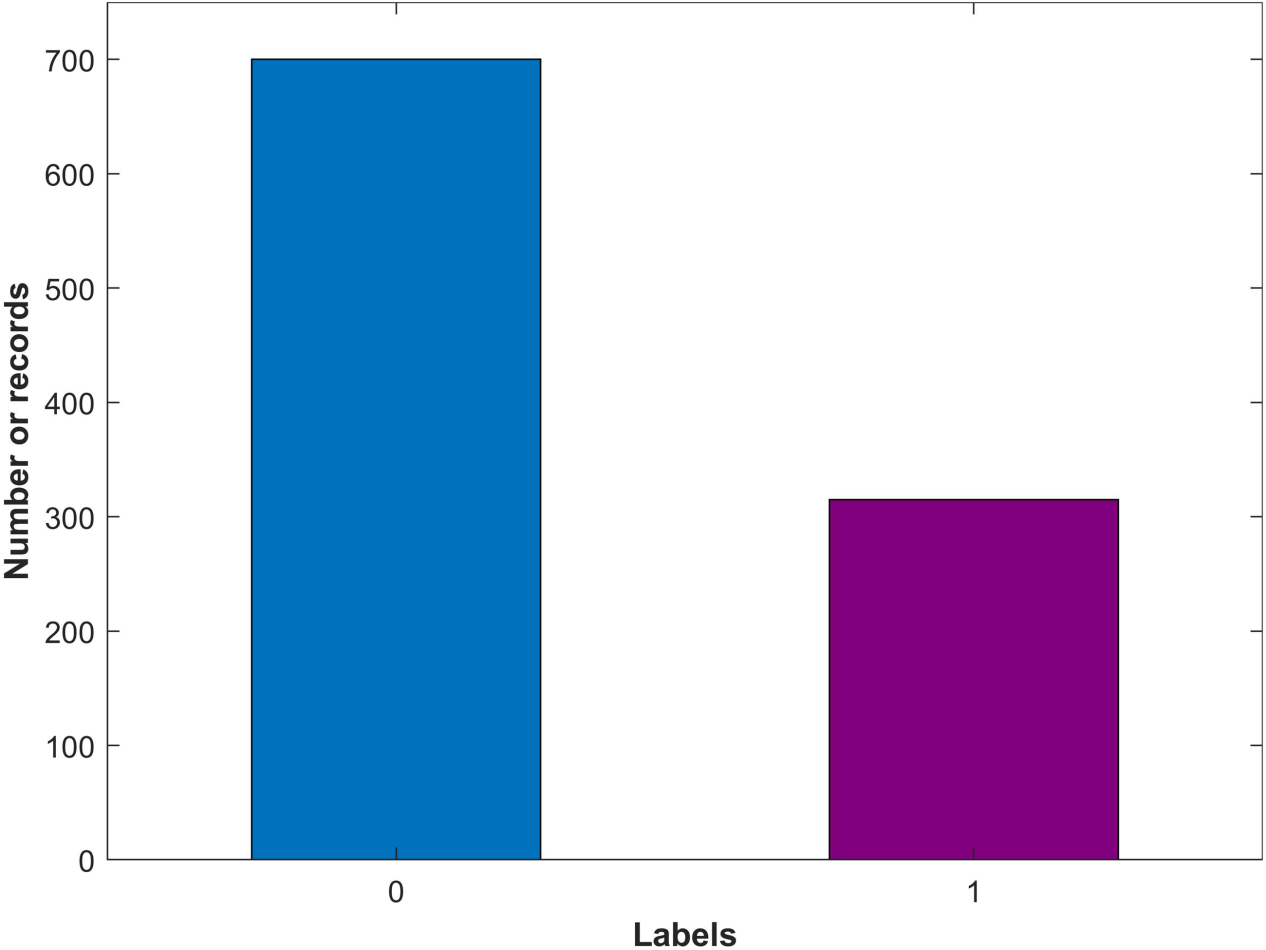
Number of records for each class in the dataset.

The collected data is preprocessed and arranged into a structured form. The dataset comprises three columns including earning calls title as ‘Title’, earning calls text as ‘text’, and the class as the Label. The label value can be ‘0’ for non-bankrupt and ‘1’ for bankrupt companies. The dataset is manually labeled by a Google search to set each company’s status whether it is bankrupted or not. Among the 1,016 companies, 311 companies are found bankrupt while the remaining 705 companies are non-bankrupt. Figure 2 shows the count for both target classes indicating that the number of samples for bankrupt and non-bankrupt companies is substantially different.

**Figure 3:**
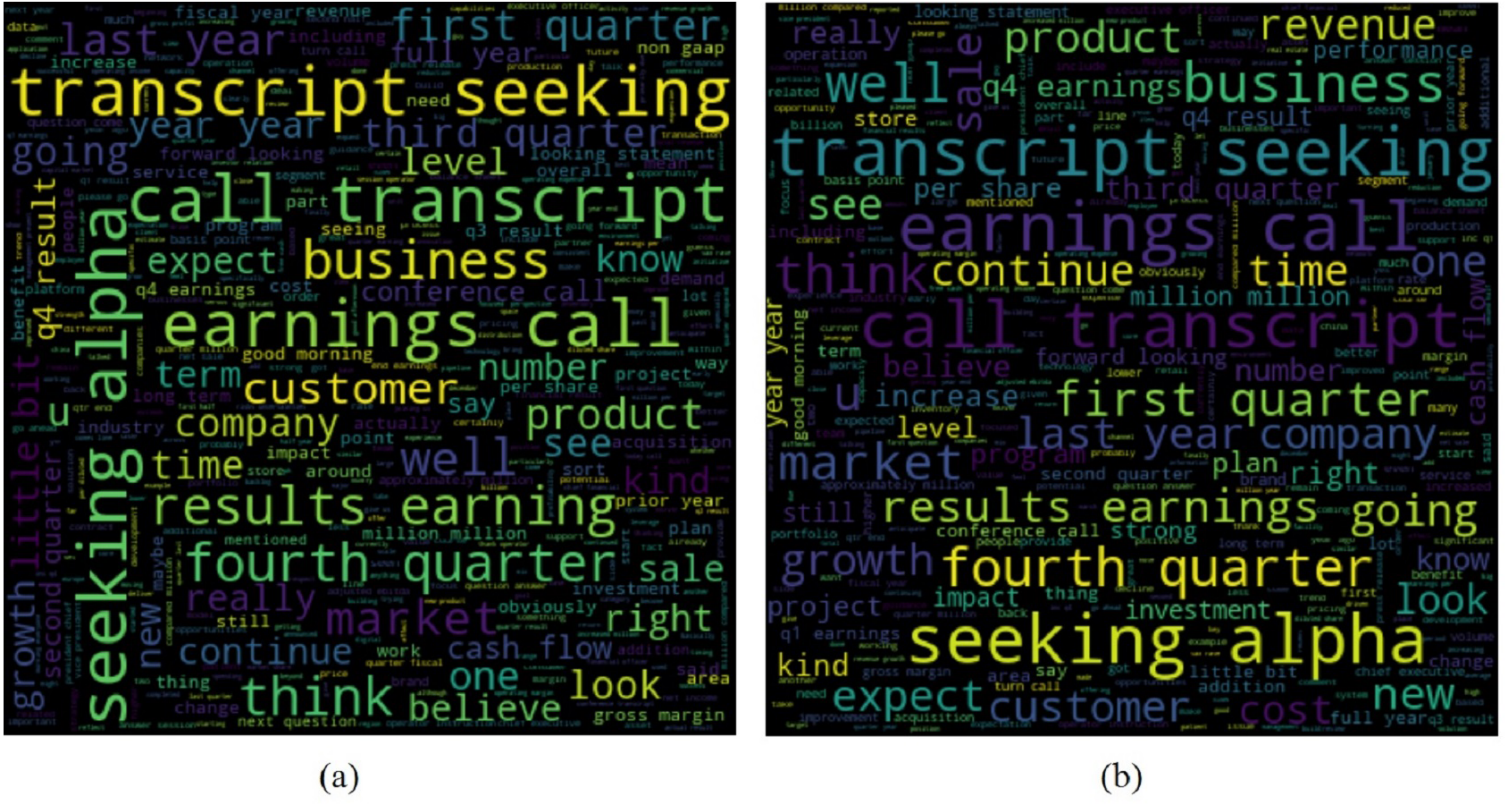
The word cloud for the dataset collected for, (A) Non-bankrupt companies, and (B) bankrupt companies.

For providing a better illustration of the text for bankrupt and non-bankrupt companies, word clouds are given in Fig. 3. Despite the similarity in the used words for both types of companies, words as well as their frequency are different for both companies and can be used to predict bankruptcy.

#### Data preprocessing

Text preprocessing/cleaning is a procedure that is used to filter out unnecessary data or noise from the raw text. Since feeding raw data to a model degrades the model’s performance, the raw data is processed before using it with the algorithms to achieve better accuracy and efficiency (Rustam et al., 2021).

As the first step, the text is converted into lowercase. Machine learning models consider ‘earning’ and ‘Earning’ as two different words which increases the complexity of the feature vector. Since the word count is an important factor in text analytics to understand the text correlation and emotion, all the words must be on the same scale to extract accurate word frequency. Case conversion ensures that all words are in a standard format which reduces the feature vector complexity and improves the performance of the models (Rustam et al., 2020a).

Special characters are non-numeric, non-alphabet letters *e.g.*, “!”, “?”, “.” *etc.* The meaning of the text document remains the same if these are removed. Each text document must have a character set indicated in it. A regular expression is used to replace with an empty string which is equivalent to all the special character that is removed from the text document (Jamil et al., 2021).

**Figure 4:**
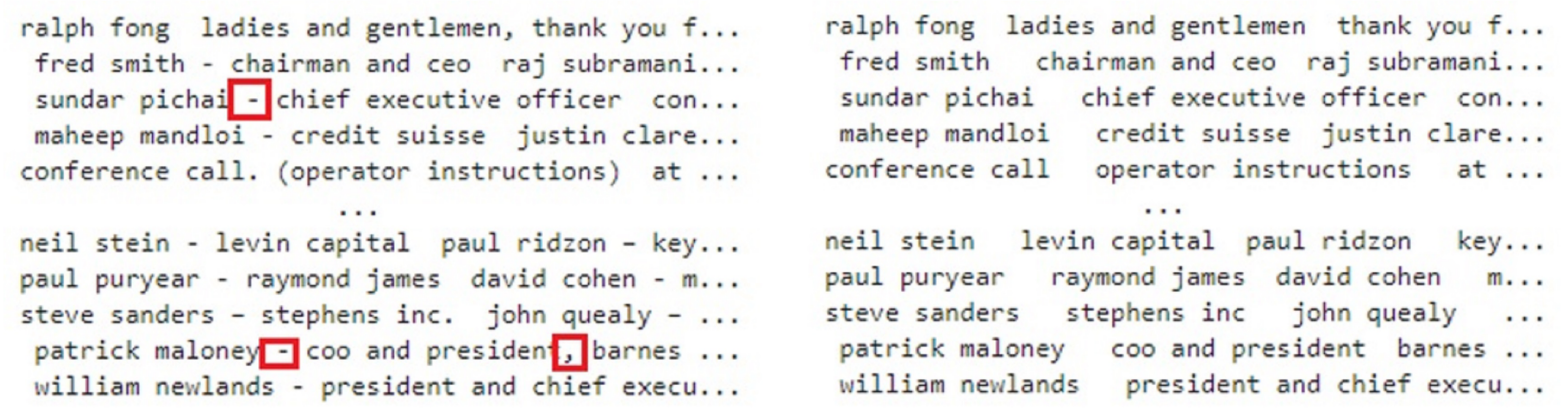
Punctuation removal from the text.

Punctuation removal is also important to reduce the complexity of model’s training. Each text document contains different punctuation characters that do not contribute to the prediction capacity of the models. The punctuation to the sentence adds up noise that brings ambiguity while training the model (Rehan et al., 2021), therefore, punctuation has been removed. Figure 4 shows the original text on the left side of the figure while the right side shows the text with removed punctuation.

**Figure 5:**
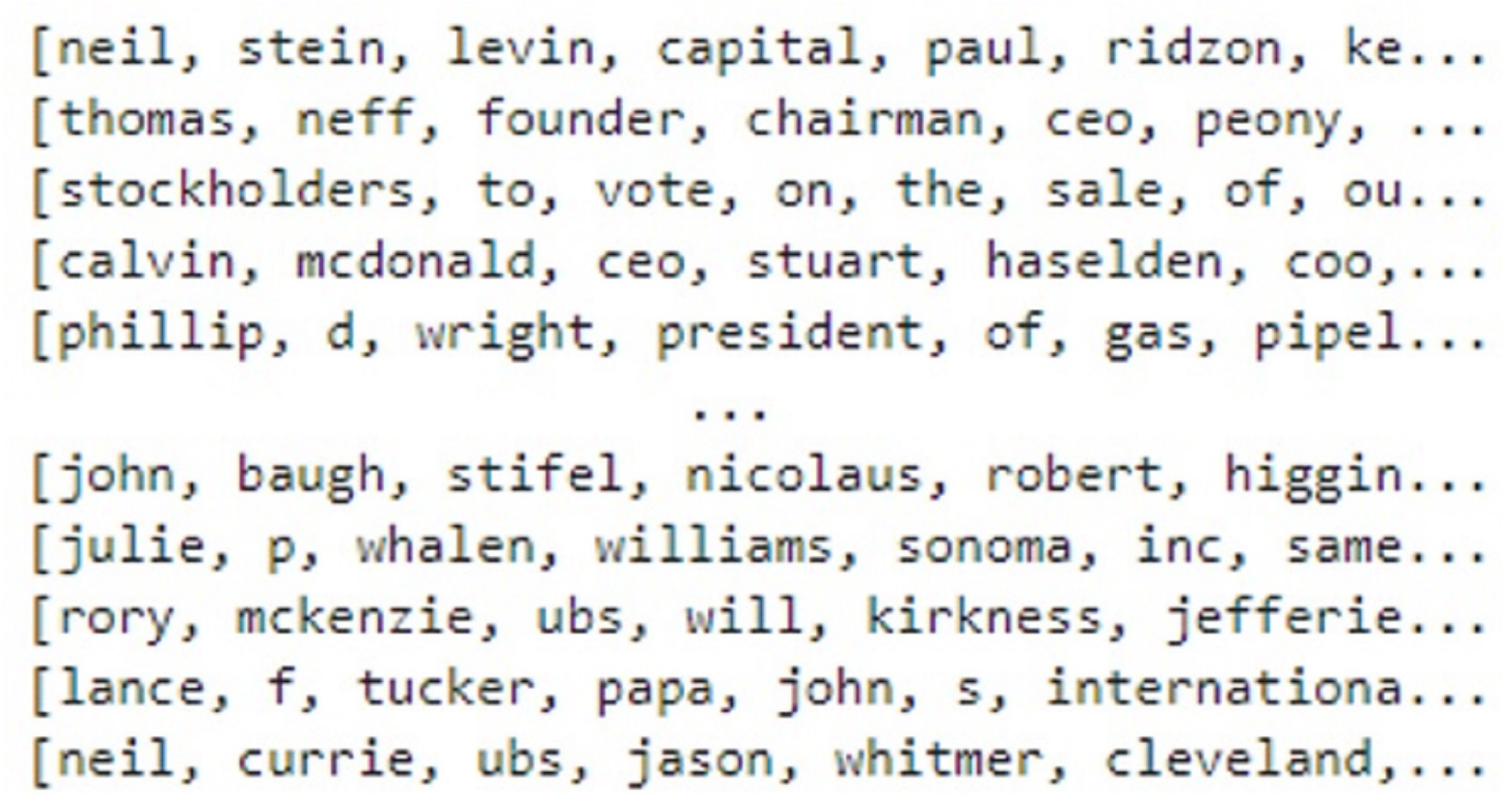
Tokenization of the text.

Tokenization is the process of separating the text into small significant units. Contingent upon the job needing to be done, tokenization isolates the information text into significant tokens. For this purpose, the text is tokenized into words as shown in Fig. 5.

Stopwords are the common words that frequently appear in the text to clarify the meanings for humans such as ‘a’, ‘the’, ‘in’, *etc.* Excluding these words from the text does not change the meaning of the sentence, instead, the complexity is reduced for machine learning algorithms (Munková, Munk & Vozár, 2013). The ‘nltk’ library is used to remove the stopwords from the text. It is followed by stemming which transforms the words into their root form. Natural language understanding (NLU) and natural language processing (NLP) both benefit from stemming. If the words are not stemmed, they are counted as different words by machine learning models which affects their performance. In this research, the stemming is performed on the textual data using the SnowballStemmer library that is a part of ‘nltk’ library (Anandarajan, Hill & Nolan, 2019).

#### Feature extraction

This study uses two feature extraction techniques including TF-IDF and BoW to make a comparison with the proposed transfer learning approach. Sklearn provides feature extraction with TF/IDF vectorizer built-in library. TF/IDF is a widely used feature extraction technique in text classification that represents the importance of words in a given corpus (Rustam et al., 2020a). It combines TF and IDF, where the former refers to the number of occurrences of a unique term in the text while the latter uses the log to assign higher weights to rare terms in the given corpus.

(1) \begin{eqnarray*}\text{TF}= \frac{\text{total number of times the word (i) appears in the documents (j)}}{\text{total number of words in documents(j)}} \end{eqnarray*}

(2) \begin{eqnarray*}\text{IDF}=\text{log} \frac{\text{Total documents}}{\text{(Total number of documents containing word (l))}} \end{eqnarray*}

(3) \begin{eqnarray*}\text{TF}-\text{IDF}=(\text{TF}\times \text{IDF}).\end{eqnarray*}

The BoW is a feature extraction technique to extract features from text data. It is a simple technique with less complexity and often produces better results than sophisticated feature extraction approaches. It extracts the frequency of words from a text document and presents them as the feature vector. We deployed this technique by using the sci-kit learn library countVectorizer (Rehan et al., 2021).

### K-Means SMOTE

The used dataset for experiments is imbalanced which increases the probability of the models’ over-fitting (Rupapara et al., 2021b). To resolve this problem we used the latest oversampling technique K-Means SMOTE which is SMOTE (Douzas, Bação & Last, 2018b). It is a combination of the K-Means cluster algorithm and SMOTE which reduces the noise and effectively overcomes the data imbalance problem within classes (Douzas, Bação & Last, 2018b).

### Classification algorithms

For classifying the companies into bankrupt and non-bankrupt based on earning calls this study used five classification models. These models are selected because of their good performance on text data, as reported in the literature. The study implements RF, ETC, ADA, SVM, and LR machine learning models for experiments that are tuned to achieve optimal accuracy using different hyperparameter settings for the classification algorithms, as shown in Table 3.

#### Random forest

It is a tree-based ensemble model used for the classification of data. RF combines the number of decision trees under majority voting criteria. Each tree predicts the sample data and these predictions pass-through voting criteria to make the final prediction (Rupapara et al., 2021b). In this study, we used 100 decision trees under RF, these decision trees will make predictions for both bankrupt and non-bankrupt classes. We used max_depth hyper-parameter with a value of 100 which restricts each tree to a maximum 100 level depth.

#### Extra tree classifier

ETC is also an ensemble model that combines multiple decision trees under majority voting criteria for the classification. The difference between RF and ETC lies in the training process. RF trains each tree on random samples from the data while in ETC each tree is trained on the whole sample data. So, RF uses bootstrap replicas while ETC uses the original samples. RF selects the best split from the trees while ETC performs it randomly. ETC also uses majority voting criteria similar to RF which makes a prediction using the predictions of all base decision trees (Rupapara et al., 2021a).

**Table 3:** Hyperparameters for machine learning models.

Classifier	Hyperparameters
RF	criterion=’entropy’, n_estimators=100, max_depth=100, random_state=None
ETC	n_estimators=100, max_depth=100, random_state=2
ADB	DecisionTreeClassifier(max_depth=100), n_estimators=100
SVM	kernel=’linear’, *C* = 1.0, cache_size=20000
LR	multi_class=ovr, Solver=liblinear, *C* = 1.0

#### AdabaBoost

ADA is an ensemble model that uses boosting criteria and combines multiple decision trees sequentially indicating that one model’s output will be input for the next tree. In this way, errors by the one decision tree will be considered by the next tree to be reduced. ADA combines the weak learners in sequence on different data. If the first weak learner wrong prediction it assigns more weight to the observation and passes to the next weak learner to make the correct prediction (Ying et al., 2013).

#### Support vector classifier

SVC is used for the classification of data. SVC draws a hyperplane to classify the data into corresponding classes. SVC draws multiple hyperplanes to classify the data and the hyperplane with the best margin from target data is considered. SVC is a linear model and we used it with the linear kernel because of the binary classification problem as it performs well for binary data (Noble, 2006).

#### Logistic regression

LR is used for the classification of data into bankrupt and non-bankrupt classes. The sigmoid function is used by the LR to classify the data into respected target classes. LR is finding the relationship between dependent and independent variables. LR is more significant with binary classification problems as in our study. We used LR with ‘liblinear’ solver which is significant on small-sized datasets (Minka, 2001).

### Proposed transfer learning model

**Figure 6:**
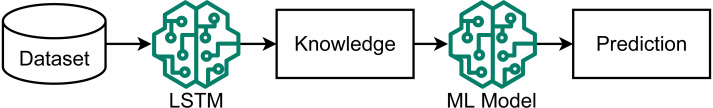
Architecture of the proposed ensemble model.

Proposed LSTM-ETC is an approach that works on the concept of transfer learning, as shown in Fig. 6. LSTM is used to extract the feature/ knowledge for the ML models. These features can be more significant for the machine learning models as compared to the traditional feature extraction techniques. This study does not use any pre-trained models in transfer learning and a custom LSTM model is built.

**Figure 7:**
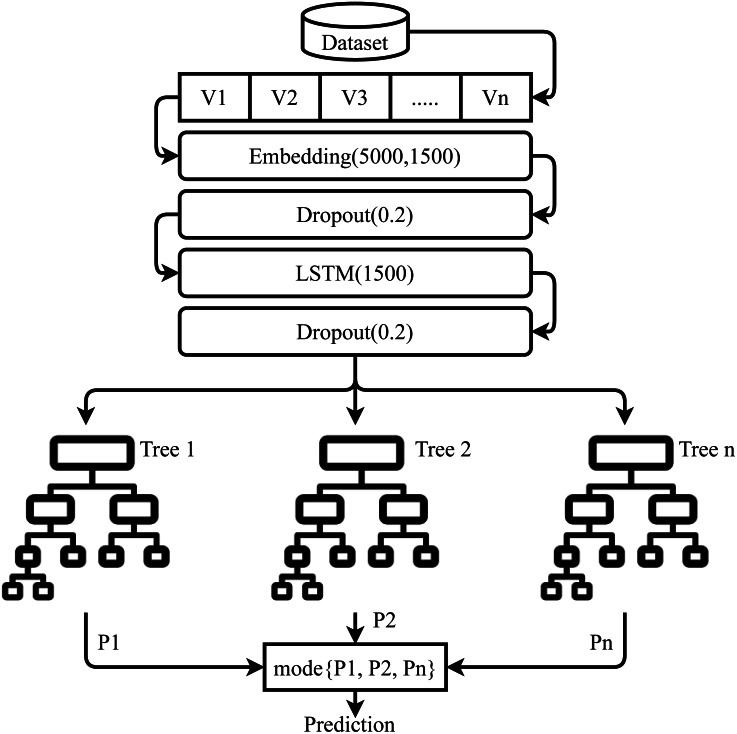
Architecture of the proposed ensemble model.

The architecture of the proposed LSTM-ETC is shown in Fig. 7. LSTM consists of five layers, with the first layer embedded with 5,000 vocabulary size and 1,500 output dimension size. The embedding layer converts the input text features into a vector which serves as the input for dropout later which is used with a 0.2 dropout rate. The dropout layer is followed by the LSTM layer with 1500 units which generates an output of 1,500 features. After this LSTM layer, another dropout layer is used with a 0.2 dropout rate to reduce complexity. The LSTM model is compiled with binary_crossentropy loss function and ‘Adam’ optimizer (Mujahid et al., 2021). The output generated by LSTM serves as the input for the ETC classifier. Each tree in ETC uses these 1,500 features for training. A total of 100 decision trees are used as the base model in ETC and each tree grows to a maximum 100-level depth. These predictions pass-through voting criteria and the class with majority voting is predicted to be the final class.

## Results and Discussions

This section contains the results of learning models for the classification of bankruptcy and non-bankruptcy target classes. The important performance evaluation parameter in this study is the F1 score because of the imbalanced dataset as it becomes important to evaluate models’ performance using an imbalanced dataset (Rustam et al., 2020b).

### Experiment setup

Experiments are performed using an Intel Core i7 11th generation machine with Windows operating system. The proposed approach is implemented using Sci-kit learn, Tensorflow, Keras, SMOTE, and NLTK libraries. Experiments are performed on the original data, as well as, the resampled data using the Kmeans SMOTE technique. For experiments, the data is split in the 0.8 to 0.2 ratios for training and testing, respectively. The number of samples for each class after the data split is shown in Table 4.

**Table 4:** Number of records for training and testing.

**Dataset**	**Class**	**Training**	**Testing**	**Total**
Original	0	573	132	705
		1	239	72	311
	Total	812	204	1,016
SMOTE	0	556	149	705
		1	572	133	705
	Total	1,128	282	1,410

### Results using BoW and TF-IDF with imbalanced dataset

Results of machine learning models on imbalanced data using BoW and TF-IDF features are shown in Table 5. The results on imbalanced data with simple BoW features are good as compared to TF-IDF. and even deep learning performance is not good on imbalanced data because, in the imbalanced dataset, the number of samples for the minority class is very small as compared to the majority class. The lack of appropriate size of the feature set with TF-IDF reduces the performance of models. The best F1 score on the imbalanced dataset with BoW and TF-IDF features is achieved by SVC which is 0.62.

**Table 5:** Results of machine learning models based on BoW and TF-IDF features using imbalanced dataset.

Model	BoW				TF-IDF			
	Accuracy	Precision	Recall	F1 score	Accuracy	Precision	Recall	F1 score
ETC	0.74	0.76	0.55	0.52	0.67	0.65	0.56	0.53
ADA	0.70	0.62	0.60	0.60	0.68	0.64	0.62	0.62
RF	0.74	0.73	0.56	0.53	0.67	0.65	0.56	0.53
LR	0.65	0.60	0.61	0.60	0.66	0.62	0.60	0.61
SVC	0.74	0.67	0.61	0.62	0.68	0.65	0.59	0.58

### Results using BoW and TF-IDF with SMOTE

Separate experiments are performed using machine learning models on the balanced data with SMOTE. Table 6 shows the results with SMOTE using BoW and TF-IDF features. Results confirm that models perform significantly better with TF-IDF features as all models achieve an accuracy score higher than 0.84 while with BoW features only RF achieves a 0.86 accuracy score. The significant accuracy score with TF-IDF features is because of weighted features as compared to BoW which gives simple term frequency as features. Data balance increased the size of the dataset and the feature vector is increased which results in a good fit of models and performance is improved.

**Table 6:** Results of machine learning models based on BoW and TF-IDF features with SMOTE-balanced dataset.

Model	BoW				TF-IDF			
	Accuracy	Precision	Recall	F1 score	Accuracy	Precision	Recall	F1 score
ETC	0.80	0.80	0.80	0.80	0.84	0.84	0.84	0.84
ADA	0.80	0.80	0.80	0.80	0.84	0.84	0.84	0.84
RF	0.86	0.86	0.86	0.86	0.84	0.84	0.84	0.84
LR	0.77	0.77	0.76	0.76	0.84	0.84	0.84	0.84
SVC	0.82	0.82	0.82	0.82	0.85	0.85	0.85	0.85

### Results using transfer learning and imbalanced dataset

Table 7 shows the results of machine learning with transfer learning on the imbalanced dataset. The results are poor because of highly imbalanced data. Models get overfitted on the majority class and give a higher number of wrong predictions for the minority class. SVC achieves a 0.72 accuracy score while its F1 score is only 0.56 which shows the difference in the model’s performance for both classes. LR performs well in terms of F1 score on imbalanced data as compared to all other used models because LR can place a higher weight on minority class in binary classification.

**Table 7:** Results using the original imbalanced dataset with transfer learning.

Model	Accuracy	Class	Precision	Recall	F1 score
ETC	0.71	0	0.91	0.96	0.82
				1	0.65	0.17	0.87
		Avg.	0.68	0.56	0.54
ADA	0.65	0	0.72	0.79	0.75
				1	0.43	0.34	0.38
		Avg.	0.58	0.56	0.57
RF	0.72	0	0.71	0.97	0.82
				1	0.73	0.17	0.28
		Avg.	0.72	0.57	0.55
LR	0.70	0	0.76	0.82	0.79
				1	0.54	0.45	0.49
		Avg.	0.65	0.63	0.64
SVC	0.72	0	0.72	0.97	0.83
				1	0.75	0.18	0.30
		Avg.	0.73	0.58	0.56

### Performance using transfer learning and SMOTE

This section contains the results of the transfer learning approach with SMOTE technique. Table 8 shows the experimental results using transfer learning with a balanced dataset. The performance of machine learning models is significantly improved with the SMOTE-balanced dataset. ETC achieves the highest accuracy score of 0.93 and an F1 score of 0.93. SVC and RF follow the ETC with 0.91 and 0.90 accuracy scores, respectively. This significant performance of ETC is because of its ensemble architecture and significant features for training provided by transfer learning. ETC is used with 200 decision trees in the prediction procedure which helps to learn significantly on neural networks generated features while SMOTE technique helps to reduce the model’s overfitting.

**Table 8:** Results using the SMOTE with transfer learning.

Model	Accuracy	Class	Precision	Recall	F1 score
ETC	0.93	0	0.90	0.97	0.93
				1	0.97	0.89	0.93
		Avg.	0.94	0.93	0.93
ADA	0.83	0	0.84	0.81	0.82
				1	0.82	0.85	0.83
		Avg.	0.83	0.83	0.83
RF	0.90	0	0.87	0.94	0.90
				1	0.94	0.86	0.90
		Avg.	0.90	0.90	0.90
LR	0.83	0	0.87	0.77	0.82
				1	0.79	0.89	0.84
		Avg.	0.83	0.83	0.83
SVC	0.91	0	0.92	0.89	0.91
				1	0.90	0.92	0.91
		Avg.	0.91	0.91	0.91

**Figure 8:**
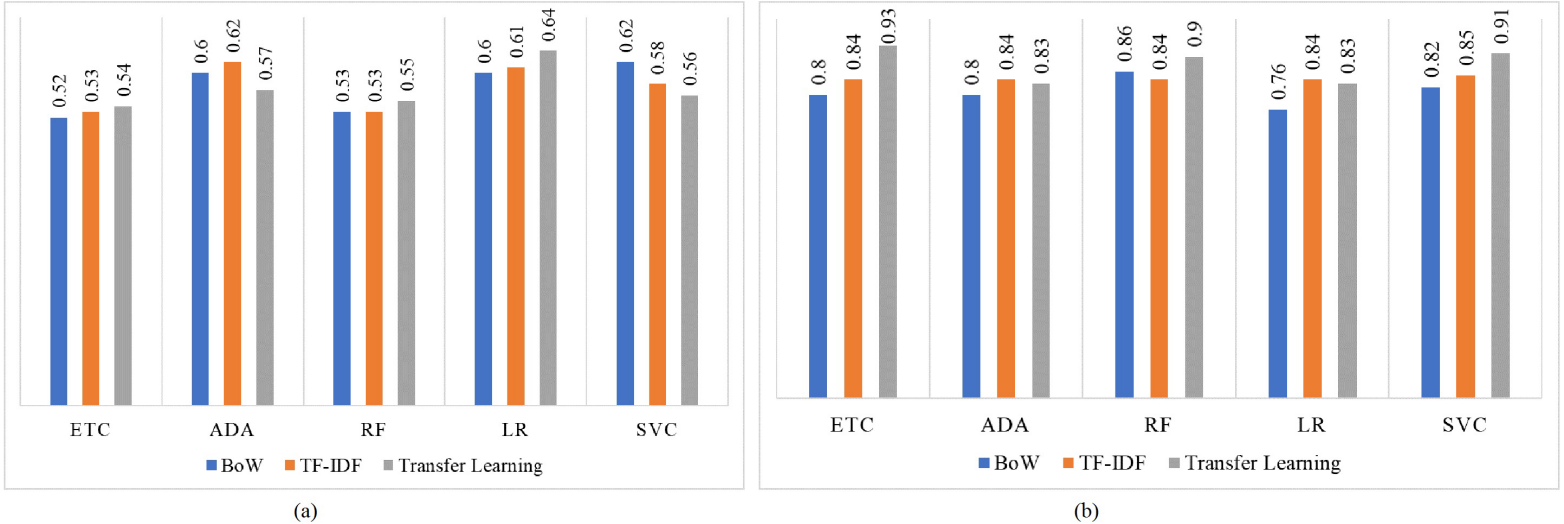
F1 score for machine learning models, (A) using the imbalanced dataset, and (B) with balanced dataset.

Figure 8 shows the F1 score for both imbalanced and balanced datasets using BoW, TF-IDF, and transfer learning features. For both imbalanced and balanced dataset cases, models perform well with transfer learning features as compared to traditional machine learning models trained using BoW or TF-IDF. LR achieves the highest F1 score of 0.64 on the imbalanced dataset while its highest F1 score using the balanced dataset is increased to 0.93 using transfer learning.

### Performance of deep learning models

For bankrupt and non-bankrupt classification, two deep learning models have also been used in addition to machine learning methods. For this purpose, LSTM, and recurrent neural network (RNN) are adopted for performance appraisal with machine learning models and transfer learning models. Models are customized in terms of neurons and layers, as well as a variety of parameters like learning rate, activation, optimization, *etc.* The parameters used for experiments and architecture of the deep learning model are given in Table 9. Each model is constructed with the ‘binary crossentropy’ loss, and the ‘Adam’ optimizer is utilized for optimization. The models are trained using 100 epochs and a batch size of 16 is used. Dropout layers with various rates are utilized in both RNN and LSTM to avoid the overfitting problem.

**Table 9:** Architecture and parameter for deep learning models.

**LSTM**	**RNN**
Embedding(5000,100)	Embedding(5000,100)
Dropout(0.2)	Dropout(0.2)
LSTM(100)	SimpleRNN (100)
Dropout(0.2)	Dropout(0.2)
Dense(2, activation=’softmax’)	Dense(2, activation=’softmax’)
Loss=‘binary_crossentropy’, optimizer= ‘adam’, epochs=100, batch_size=16	

The results of the deep learning model are shown in Table 10 indicating that deep learning models perform poorly as compared to machine learning models because of the small size of the dataset as deep learning models require a large dataset for significant performance. We deployed deep learning models on both balanced and imbalanced datasets. RNN obtains the best F1 score of 0.48 on the imbalanced dataset as compared to LSTM with a 0.42 F1 Score. The performance of LSTM is improved after balancing the dataset with a 0.60 F1 score because oversampling increases the dataset size. Primarily, the lack of an appropriate dataset in terms of size resulted in the poor performance of these models. Deep learning models are data-intensive and often require a large size dataset to learn the complex correlations which is not the case for the current study.

**Table 10:** Performance of deep learning models.

Approach	Model	Accuracy	Precision	Recall	F1 score
Imbalanced	LSTM	0.73	0.37	0.50	0.42
	RNN	0.60	0.49	0.50	0.48
Balanced	LSTM	0.61	0.60	0.60	0.60
	RNN	0.48	0.50	0.50	0.43

### *T*-test results

To show the significance of the proposed approach LSTM-ETC, we perform a statistical *T*-test. We perform a *T*-test between models’ performance using transfer learning and TF-IDF and transfer learning and BoW. We deploy the *T*-test with a 0.5 alpha and the output of the *T*-test is evaluated in terms of t-statistic (t), degrees of freedom (df), and critical value (CV), as shown in Table 11.

**Table 11:** Statistical significance *T*-test results.

Comparison	t	df	CV	Null Hypothesis
TL vs TF-IDF	3.853	4	7.06 e^−^17	Rejected
TL vs BoW	1.868	4	7.06 e^−^17	Rejected

Results of the *T*-test show that for both cases transfer learning Versace TF-IDF and transfer learning Versace BoW, the null hypothesis is rejected because *t* is less than CV which means that transfer learning results are significant as compared to TF-IDF and BoW results.

### K-fold cross validation

The 10-fold cross-validation is performed to corroborate the efficiency of the proposed approach. Table 12 shows the results of cross-validation. All models perform better when used with SMOTE balanced data, however, the transfer learning approach and ETC achieve the highest mean accuracy with 0.09 standard deviation (SD). It is followed by the RF model which has a 0.85 mean accuracy and 0.10 SD. Results show that the tree-based ensemble models perform better than other models with the used dataset.

**Table 12:** 10 fold cross validation results using the SMOTE with transfer learning.

Model	Accuracy	SD
ETC	0.86	+/ −0.09
ADA	0.76	+/ −0.10
RF	0.85	+/ −0.10
LR	0.76	+/ −0.06
SVC	0.83	+/ −0.07

### Performance comparison with existing studies

This study compares the performance of the proposed approach with existing studies on bankruptcy prediction. Table 13 shows the performance comparison. It can be observed that the performance of the proposed approach is better than existing studies.

## Conclusions and Future Work

Businesses can collapse in both small and big economies due to different financial, managerial, social, economic, and social factors. A bad managerial decision, new economic and environmental policy, an uncommon debt, and a social clash can lead to a firm’s bankruptcy. The well-being of a firm directly influences many stakeholders like creditors, investors, shareholders, *etc.* and prediction regarding a firm’s upcoming financial collapse can prepare the management and investors to prepare and execute contingency plans. Predominantly, prediction approaches and frameworks rely on the financial indicators from the stock exchange and banks for predicting bankruptcy however, such indicators represent past data and the status of firms’ current operations are not revealed. Analyzing the textual data and quantifying the sentiment of financial disclosure, emotion, and tone of a CEO in the meeting can be used. The earning calls data has plentiful information and can be used to predict the bankruptcy of a firm. This study proposes a transfer learning-based model in this regard and experiments with a self-collected and manually labeled dataset. Features extracted from the LSTM model are used to train the machine learning models on the original and K-Means SMOTE balanced datasets. Experimental results prove the superior performance of the proposed approach where the ETC model achieves the highest accuracy and F1 score of 0.93 with the balanced dataset. The performance of deep learning models is poor due to the small data size. Statistical *t*-Test and cross-validation corroborate the significant performance of the proposed approach as well. We would like to extend the experiments with an updated and large-sized dataset in the future.

**Table 13:** Comparison with previous studies.

Reference	Dataset	Classifier	Performance
Kasgari, Salehnezhad & Ebadi (2013)	Own Dataset	MLP, LR	0.87 and 0.90 Accuracy
Wang et al. (2013)	Dataset taken from Kogan	Regression (LOG1P+), Ranking (TFIDF+)	0.14894 MSE, 0.15271 MSE, 0.62939 Spearman’s Rho , 0.63403 Spearman’s Rho
Rönnqvist & Sarlin (2017)	Own Dataset	NN	*σ* = 0.008
Qin & Yang (2019)	Text and Audio Dataset	MDRM	Text + Audio = 0.217 MSE
Current study	Own dataset	ETC, ADA, RF, LR, SVC	0.93, 0.83, 0.90, 0.83, and 0.91 Accuracy

##  Supplemental Information

10.7717/peerj-cs.1134/supp-1 Supplemental Information 1 Code for machine learning models

10.7717/peerj-cs.1134/supp-2 Supplemental Information 2 Raw data

## Additional Information and Declarations

### Data Availability

The following information was supplied regarding data availability: The machine learning implementation code and dataset are available in the Supplementary File.
